# Long-term efficacy of hydrotherapy on balance function in patients with Parkinson’s disease: a systematic review and meta-analysis

**DOI:** 10.3389/fnagi.2023.1320240

**Published:** 2023-12-13

**Authors:** Zicai Liu, Miao Huang, Ya Liao, Xiuying Xie, Pingan Zhu, Yangyou Liu, Cheng Tan

**Affiliations:** ^1^Department of Rehabilitation Medicine, Shaoguan First People’s Hospital, Shaoguan, China; ^2^School of Rehabilitation Medicine Gannan Medical University, Ganzhou, China

**Keywords:** hydrotherapy, Parkinson’s disease, balance, meta-analysis, PD

## Abstract

**Background:**

Hydrotherapy can improve the motor and non-motor symptoms of Parkinson’s disease (PD), but the long-term effects of hydrotherapy on PD are still unclear.

**Objective:**

The purpose of this systematic evaluation and meta-analysis was to explore the long-term effects of hydrotherapy on balance function in PD patients.

**Methods:**

A systematic search of five databases was conducted to identify appropriate randomized controlled trials (RCTs) according to the established inclusion and exclusion criteria. The general characteristics and outcome data (balance, exercise, mobility, quality of life, etc.) of the included studies were extracted, and the quality of the included studies was evaluated using the Cochrane risk of bias assessment tool. Finally, the outcome data were integrated for meta-analysis.

**Results:**

A total of 149 articles were screened, and 5 high-quality RCTs involving 135 PD patients were included. The results of the meta-analysis showed positive long-term effects of hydrotherapy on balance function compared to the control group (SMD = 0.69; 95% CI = 0.21, 1.17; *p* = 0.005; *I*^2^ = 44%), However, there were no significant long-term effects of hydrotherapy on motor function (SMD = 0.06; 95% CI = −0.33, 0.44; *p* = 0.77; *I*^2^ = 0%), mobility and quality of life (SMD = −0.21; 95% CI = −0.98, 0.57; *p* = 0.6; *I*^2^ = 71%). Interestingly, the results of the sensitivity analysis performed on mobility showed a clear continuation effect of hydrotherapy on mobility compared to the control group (SMD = −0.80; 95% CI = −1.23, −0.37; *p* < 0.001; *I*^2^ = 0%).

**Conclusion:**

The long-term effects of hydrotherapy on PD patients mainly focus on balance function, and the continuous effects on motor function, mobility, and quality of life are not obvious.

## 1 Introduction

Parkinson’s disease (PD) is a neurodegenerative disorder characterized by progressive worsening of motor and non-motor dysfunction (Sveinbjornsdottir, 2016). Epidemiologic studies show a global incidence of PD of 200/100,000 people, with one person diagnosed approximately every hour (Titova and Chaudhuri, 2018). Additionally, the incidence increases 5 to 10 times from the age of 60 to 90 years, resulting in an increase of 5/100,000 to more than 35/100,000 new cases per year (Simon et al., 2020). Disease progression is associated with limited mobility, increased risk of falls, and decreased quality of life (Hariz and Forsgren, 2011). Impaired balance is a common symptom that increases the risk of falls in people with PD (Simpkins and Yang, 2023). As PD progresses, the processing of vestibular, visual, and proprioceptive signals that maintain body balance changes (Samoudi et al., 2015). PD patients tend to move their center of gravity forward, making it difficult for them to perform compensatory movements and adjustments of such body balance, which leads to more frequent falls (Samoudi et al., 2015).

The treatment of PD includes drug therapy and physical therapy (Poewe and Seppi, 2001; Reinoso et al., 2015). Medication can relieve PD symptoms, but it does not change the progression of the disease and has some side effects (Reinoso et al., 2015). For example, levodopa may cause side effects such as nausea, delusions, drowsiness, and dystonia (Hayes, 2019). Physical therapy is used by an increasing number of scholars and physicians as an adjunct to PD treatment (Gage and Storey, 2004). Physical therapy for PD focuses on transfers, posture, upper extremity function, balance (and falls), gait, and physical ability and (in) mobility (Tomlinson et al., 2012). Hydrotherapy as a physical therapy for the treatment of motor and non-motor symptoms in patients with early-stage PD has been shown to have favorable results in terms of slowed movement, dystonia, balance, pain, quality of life, and physical function (Alves Da Rocha et al., 2015; Carroll et al., 2020, 2022). The aquatic environment has specific mechanical advantages due to hydrostatic and hydrodynamic principles of buoyancy, viscosity, and resistance (Denning et al., 2012). Specific properties of the aquatic environment (density, specific gravity, hydrostatic pressure, buoyancy, viscosity, and thermodynamics) may help to modulate sensory feedback of motor output (Cugusi et al., 2019). Buoyancy reduces weight and allows the patient to transfer safely at a lower speed (Carregaro and AMd, 2008). Due to its ability to enhance functional mobility, the effect on PD patients at the same time is pleasant. Hydrotherapy has become a popular treatment (Cugusi et al., 2015). The current meta-analysis reports that hydrotherapy has positive outcomes in gait, balance, and mobility, but only analyzes efficacy during treatment and does not explore the long-term effects of hydrotherapy on PD patients or (how long these improvements can be sustained?) (Carroll et al., 2020). Therefore, the purpose of this systematic evaluation and meta-analysis was to explore the long-term effects of hydrotherapy on balance function in PD patients.

## 2 Methods

### 2.1 Protocol and registration

This systematic review was designed and implemented by the Preferred Reporting Items for Systematic Reviews and Meta-analysis (PRISMA) guidelines (Page et al., 2021). The study was registered in PROSPERO (CRD42023465857).

### 2.2 Search strategy

Searches were conducted in five databases (PubMed, Cochrane Library, Embase, Web of Science, and Ovid) by the 2020 PRISMA statement (Page et al., 2021). The search was limited to September 24, 2023. The search strategy in the Pubmed database uses subject terms paired with keywords for the search. For example: [(“Hydrotherapy”[Mesh]) OR (Whirlpool Baths) OR (Bath, Whirlpool)] AND [(“Parkinson Disease, Secondary”[Mesh]) OR (Parkinson Disease) OR (Secondary Parkinson Disease) OR (Parkinsonism, Symptomatic)].

### 2.3 Inclusion and exclusion criteria

The criteria for inclusion in the study were: (1) The study population were PD patients (Hoehn and Yahr I–IV); (2) Intervention was hydrotherapy (or physical therapy or exercise was done in a pool setting); (3) The interventions in the control group was land-based treatments or routine exercises; (4) The outcome indicators included in the study must contain the Berg Balance Scale (BBS). In addition, the patient’s motor function, mobility, or quality of life will be evaluated (meeting one of these is sufficient); (5) Follow-up assessment was mandatory for the included studies; (6) The type of study was limited to randomized controlled trials (RCTs). (6) Exclusion Criteria: (1) Follow-up time from the end of the intervention was too short, less than 15 days; (2) Data could not be extracted from the included studies.

Two researchers (ZCL and MH) assessed the studies separately. First, they eliminate duplicates and then evaluate the titles and abstracts of the remaining literature. Finally, studies that met the criteria were read in full text. All differences were discussed and resolved by two researchers.

### 2.4 Data extraction

Two researchers (ZL and MH) independently evaluated the studies based on the above inclusion/exclusion criteria. They extract data as well as information in two parts. The first part is the basic information about the included studies, including authors, year of publication, type of study design, intervention program, and assessment indicators. The second part is the extracted data such as the number of people, age, duration of follow-up, and assessment data. All differences were discussed and resolved by two researchers.

### 2.5 Quality assessment

Two researchers (XX and CT) independently evaluated the quality of the method and the risk of bias included in the study through the Cochrane Bias Risk Tool and checked seven items including potential selection bias, implementation bias, detection bias, loss bias, reporting bias, and other biases. The tool divides quality risk into three categories: low, high, and unclear.

### 2.6 Data synthesis and statistical analysis

RevMan5.4.1 statistical software is used for data analysis. Standardized mean difference (SMD) of clinical scale change scores and their 95% confidence intervals were used for calculation. This study explored the long-term effects of hydrotherapy on PD patients, so we used the values of change in clinical scores from baseline to follow-up. 95% confidence intervals (CIs) were calculated using the z-test. Heterogeneity between groups was tested using the *p*-value test and I^2^ test. Heterogeneity between studies was indicated if *p* < 0.05 and *I^2^* > 50%. Sensitivity analysis was performed to ensure the reliability of the results.

## 3 Results

### 3.1 Literature search and study characteristics

**Figure 1:**
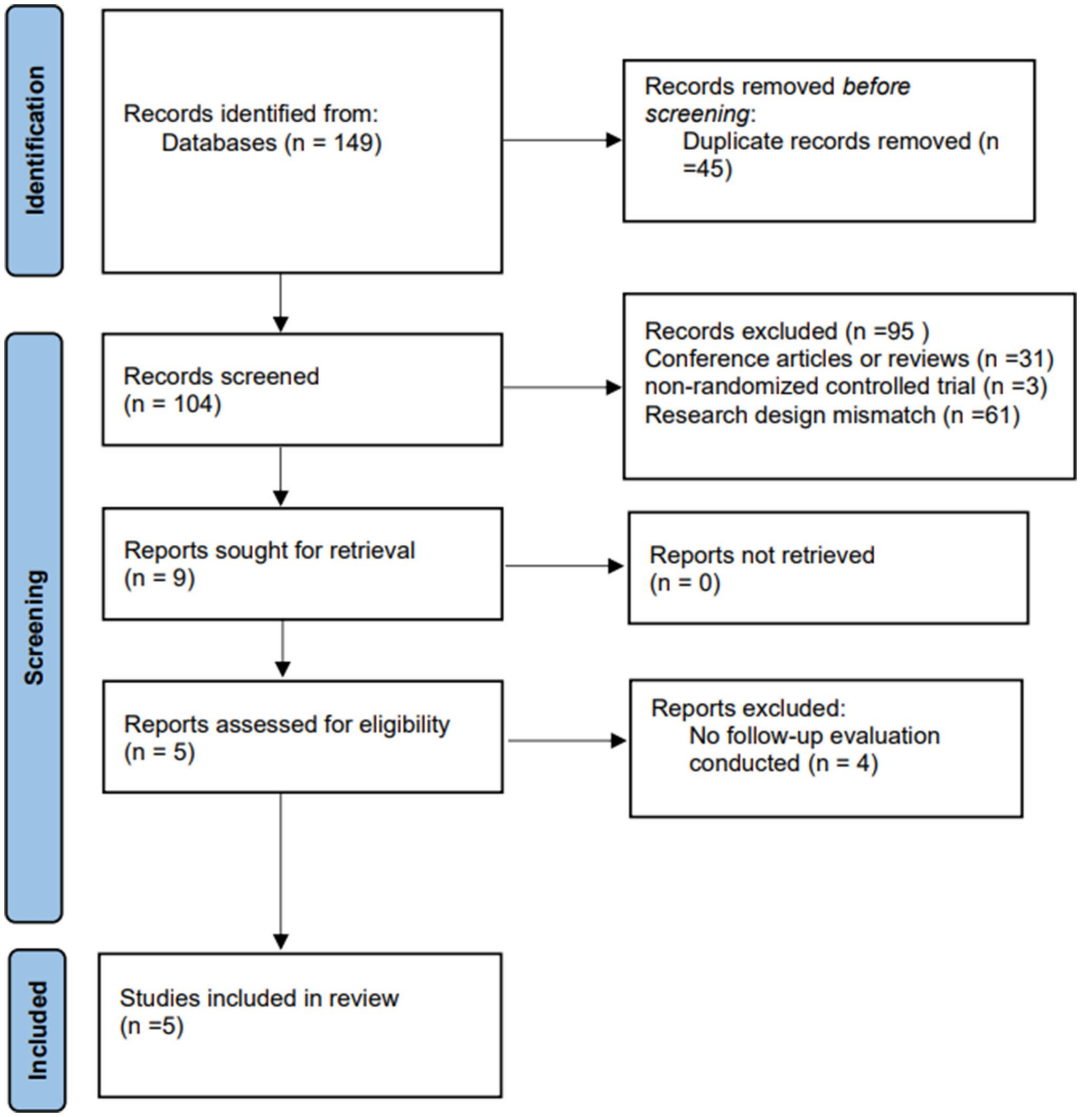
PRISMA flow chart of study selection.

This study initially searched five databases and screened 149 potential studies, 43 of which were duplicates. We then screened the remaining 104 studies for titles and abstracts, 95 studies were excluded, and 9 studies were assessed in full text. Ultimately, only five studies were included in the analysis. The specific screening process is shown in Figure 1.

As shown in Table 1, a total of 5 studies including 135 PD patients were included. All five studies (Vivas et al., 2011; Palamara et al., 2017; Pérez de la Cruz, 2017; Volpe et al., 2017; Da Silva and Israel, 2019) were RCTs by design, but four of them were parallel randomized controlled trials (Vivas et al., 2011; Palamara et al., 2017; Pérez de la Cruz, 2017; Volpe et al., 2017), and one (Da Silva and Israel, 2019) was a non-equivalent randomized controlled trial. The smallest sample size included in the study was only 12 patients and the largest was 34 patients (Vivas et al., 2011; Palamara et al., 2017). Interventions for the control group included in the study included land-based therapy (Vivas et al., 2011), non-aquatic physical therapy (Volpe et al., 2017), intensive multidisciplinary rehabilitation (Palamara et al., 2017), dryland therapy (Pérez de la Cruz, 2017), and regular exercise (Da Silva and Israel, 2019). All five studies (Vivas et al., 2011; Palamara et al., 2017; Pérez de la Cruz, 2017; Volpe et al., 2017; Da Silva and Israel, 2019) used BBS to assess balance function in patients with PD. Four studies (Vivas et al., 2011; Palamara et al., 2017; Pérez de la Cruz, 2017; Volpe et al., 2017) used the Unified Parkinson’s Disease Rating Scale III (UPDRS III) to assess motor function in patients with PD. Four studies (Palamara et al., 2017; Pérez de la Cruz, 2017; Volpe et al., 2017; Da Silva and Israel, 2019) used the Timed Up and Go Test (TUG) to assess mobility in patients with PD. Three studies (Palamara et al., 2017; Pérez de la Cruz, 2017; Volpe et al., 2017) assessed the quality of life of patients with PD, two of which used the Unified Parkinson’s Disease Rating Scale II (UPDRS II)(Palamara et al., 2017; Pérez de la Cruz, 2017) and one of which used the Parkinson’s disease quality of life questionnaire-39 items (PDQ-39, 24].

**Table 1:** Characteristics of the included studies timed up and go test.

Author (year)	Random type	Age (years) (M ± SD)	Group (*N*)	Intervention	Follow-up time	Outcome
Vivas et al. (2011)	Simple randomization	E:65.67 ± 3.67\C:68.33 ± 6.92	E:6\C:6	E: water-based therapy C: land-based therapy	17 days after the intervention	UPDRS-III BBS
Volpe et al. (2017)	Simple randomization	E: 70.6 ± 7.8\C: 70.0 ± 7.8	E:15\C:15	E: water-based physiotherapy C: non-water- based physiotherapy	2 months after the intervention	UPDRS-III PDQ-39 BBS TUG
Palamara et al., 2017	Simple randomization	E: 70.9 ± 5.7\C: 70.8 ± 5.3	E:17\C:17	E: MIRT+AT C: MIRT	6 months after discharge	UPDRS-III UPDRS-II BBS TUG
Pérez de la Cruz, (2017)	Stratified randomization	E: 63.12 ± 13.61\C: 64.23 ± 13.45	E:15\C:15	E: aquatic therapy C: dry land therapy	1 month after discharge	UPDRS-III UPDRS-II BBS TUG
Da Silva and Israel (2019)	Simple randomization	E:66.8 ± 5.26\C: 67.53 ± 9.89	E:14\C:15	E: aquatic therapy C: regular exercise	3 months after discharge	BBS TUG

M ± SD, mean ± Standard deviation; E, Experimental group; C, Control group UPDRS-III, the Unified Parkinson’s Disease Rating Scale III; UPDRS-II, the Unified Parkinson’s Disease Rating Scale II; BBS, the Berg Balance Scale; TUG, the Timed Up and Go Test; PDQ-39, Parkinson’s disease quality of life questionnaire-39 items; MIRT, Multidisciplinary Intensive Rehabilitation Treatment; AT, aquatic therapy.

All five studies (Vivas et al., 2011; Palamara et al., 2017; Pérez de la Cruz, 2017; Volpe et al., 2017; Da Silva and Israel, 2019) were evaluated at follow-up, which ranged from a minimum of 17 days to a maximum of 6 months (Vivas et al., 2011; Palamara et al., 2017).

### 3.2 Risk of bias

**Figure 2:**
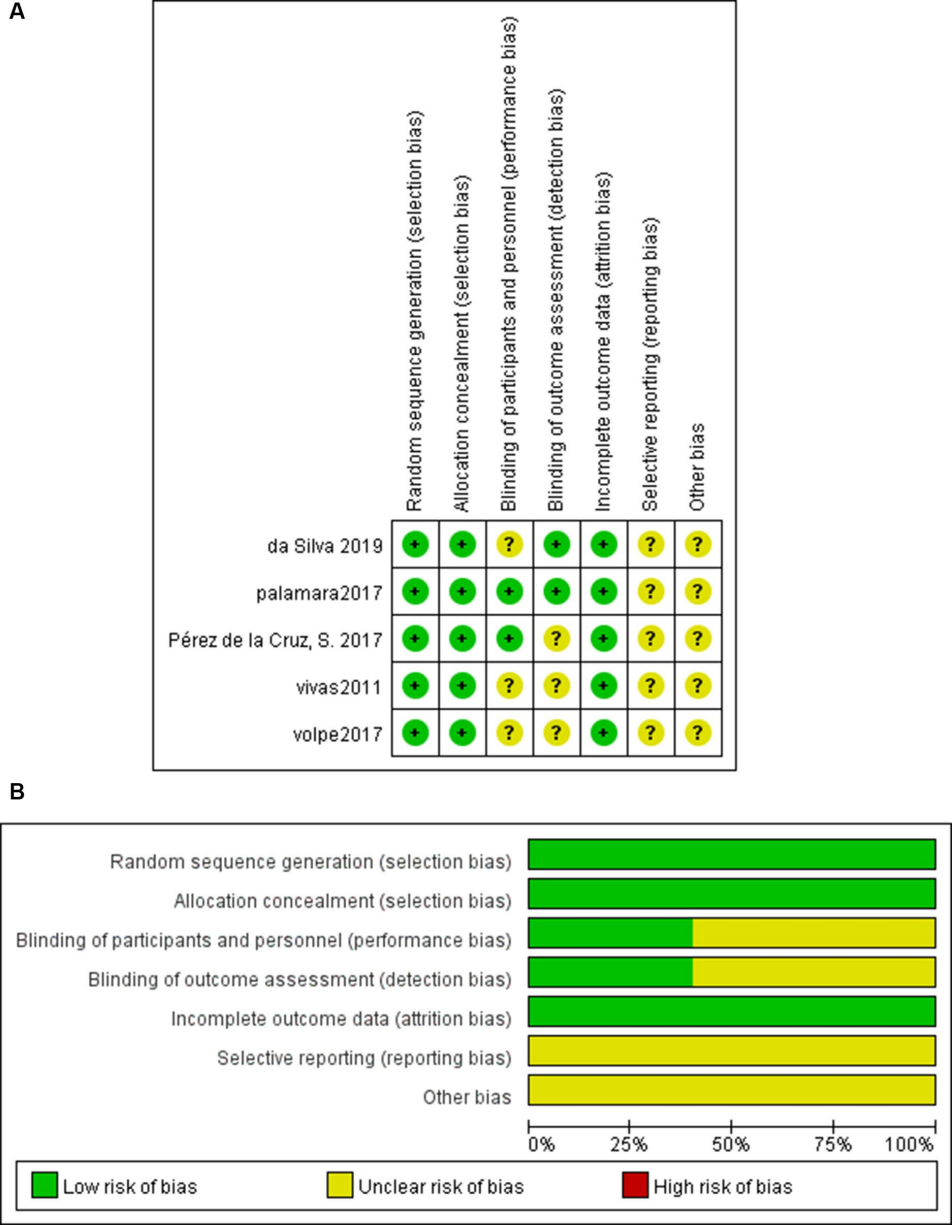
**(A)** Risk of bias graph. **(B)** Risk of bias summary.

The results of the bias risk plot included in the study are shown in Figures 2A,B. The included articles showed low-risk random sequence generation (100%), allocation concealment (100%), Blind outcome assessment (40%), incomplete outcome data (100%), selective reporting (100%), and other biases (100%). Due to the particularity of intervention measures, the low risk of blinding of participants and personal ratio (40%), uncertain risk (60%). The percentage of other bias uncertainty is 100%.

### 3.3 Long-term effects of hydrotherapy on balance function

**Figure 3:**
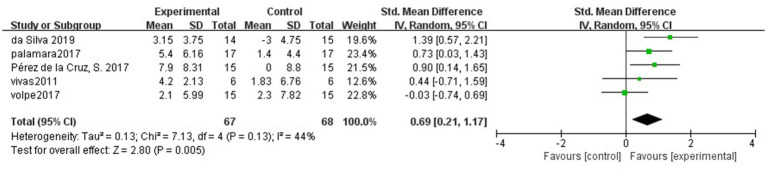
Long-term effects of hydrotherapy in balance function.

All five included studies (Vivas et al., 2011; Palamara et al., 2017; Pérez de la Cruz, 2017; Volpe et al., 2017; Da Silva and Israel, 2019) used BBS to follow balance function in patients with PD. The effect of the hydrotherapy group on balance function improvement could be maintained for a longer period compared to the control group (SMD = 0.69; 95% CI = 0.21, 1.17; *p* = 0.005; *I^2^* = 44%; Figure 3).

### 3.4 Long-term effects of hydrotherapy on motor function

**Figure 4:**
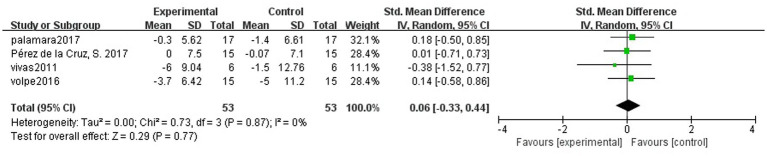
Long-term effects of hydrotherapy in motor function.

Four studies (Vivas et al., 2011; Palamara et al., 2017; Pérez de la Cruz, 2017; Volpe et al., 2017) of balance function in PD patients were followed. There was no significant difference in the maintenance of motor function in PD patients in the hydrotherapy group compared to the control group (SMD = 0.06; 95% CI = -0.33, 0.44; *p* = 0.77; *I^2^* = 0%; Figure 4).

### 3.5 Long-term effects of hydrotherapy on mobility

**Figure 5:**
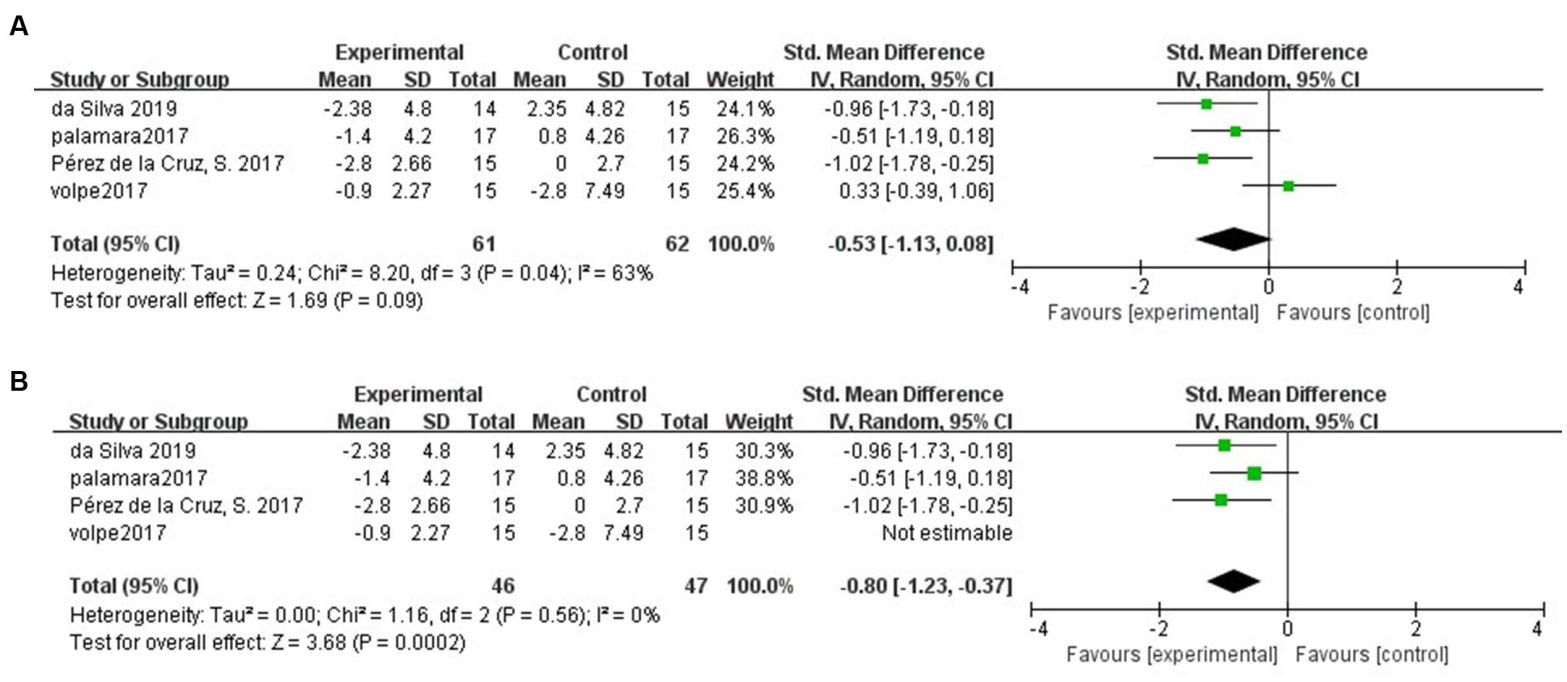
**(A)** Long-term effects of hydrotherapy in mobility. **(B)** Long-term effects of hydrotherapy in mobility (Sensitivity analysis).

Four of the included studies (Vivas et al., 2011; Palamara et al., 2017; Pérez de la Cruz, 2017; Da Silva and Israel, 2019) assessed the mobility of PD patients by TUG at follow-up. The results showed no difference in the maintenance of mobility in PD patients in the hydrotherapy group compared to the control group (SMD = −0.53; 95% CI = −1.13, 0.08; *p* = 0.09; *I^2^* = 63%; Figure 5A). The results yielded greater than 50% heterogeneity between studies, so we performed a case-by-case sensitivity analysis. The results showed that excluding the Volpe et al. study (Volpe et al., 2017) not only significantly reduced heterogeneity but also yielded inconsistent results (SMD = -0.80; 95% CI = −1.23, −0.37; *p* < 0.001; *I^2^* = 0%; Figure 5B).

### 3.6 Long-term effects of hydrotherapy on quality of life

**Figure 6:**
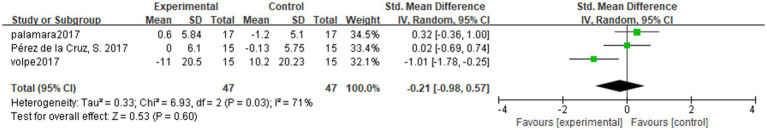
Long-term effects of hydrotherapy on quality of life.

Three studies (Palamara et al., 2017; Pérez de la Cruz, 2017; Volpe et al., 2017) have evaluated the quality of life of PD patients at follow-up. The meta-analysis showed: The results of the meta-analysis showed that there was no significant difference in the maintenance of the quality of life in PD patients in the hydrotherapy group compared to the control group (SMD = -0.21; 95% CI = -0.98, 0.57; *p* = 0.6; *I^2^* = 71%; Figure 6). Results after performing sensitivity analyzes showed no significant change in heterogeneity or outcome.

## 4 Discussion

The studies included in this study all evaluated PD patients at follow-up. The results of the meta-analysis showed that hydrotherapy had a more positive maintenance effect on balance function compared to land-based treatment, but no positives were seen in motor function, mobility, or quality of life.

### 4.1 Long-term effects of hydrotherapy on balance

The results of the analysis showed that hydrotherapy had a positive long-term effect on balance function compared with the control group. The impaired balance function in PD patients is altered processing of proprioceptive information, possibly due to the link between dysfunction of basal ganglia circuits and altered integration of peripheral inputs (Carpenter and Bloem, 2011; Conte et al., 2013). It has also been hypothesized that postural deformities in PD may also be associated with somatosensory integration dysfunction (Doherty et al., 2011).

It has been shown that the water environment can act on peripheral sensory receptors to stimulate the proprioceptive system in the balance control system (Schoneburg et al., 2013). It has been shown that the aquatic environment can increase proprioceptive input to the immersed body, resulting in better body alignment (Massion et al., 1995). In addition to this, the specific nature of the water environment plays an important role in improving and maintaining the equilibrium process (Volpe et al., 2017).

First, the hydrostatic pressure of the water supports the submerged body, providing a gravity-reducing environment that reduces the risk of falling (Caromano, 2002; Carregaro and AMd, 2008). Secondly, the buoyancy of water reduces the burden on the joints, thus improving their dynamic flexibility (Caromano, 2002). When hydrotherapy is combined with temperature, it can reduce pain and stiffness (Hinman et al., 2007; Hall et al., 2008). Thirdly, the viscosity of the water then provides a natural resistance to body movement, facilitating different exercise training as well as activating reactive postural adjustments in PD patients (Schoneburg et al., 2013; Cugusi et al., 2019). These characteristics of the aquatic environment allow some people with postural instability, high risk of falls, leg weakness, and gait disorders to exercise successfully in situations that would not be feasible or safe on land (Batterham et al., 2011). And finally, some findings suggest that balance-related training has the longest continuation effect compared to other training (Mak et al., 2017). The characteristics, as well as the benefits of hydrotherapy, seem to explain the positive long-term effects of hydrotherapy on homeostatic functioning in PD patients.

### 4.2 Long-term effects of hydrotherapy on motor function, mobility and quality of life

Previous studies have demonstrated that hydrotherapy improves motor function, mobility, and quality of life in PD patients (Carroll et al., 2017; Di Marco et al., 2022). However, no studies have examined the long-term effects of hydrotherapy. The results of this study showed no positive long-term effects of hydrotherapy on motor function, mobility, and quality of life compared to the control group. PD is a neurodegenerative disease with persistent impairment of motor function (Pringsheim et al., 2014), such as muscle weakness as well as decreased aerobic capacity, and gait disturbance (Allen et al., 2009; Nocera et al., 2010). However, some studies have established that exercise and physical therapy can change motor function in PD, but only if long-term training is achieved (Mak et al., 2017). In addition, maintaining a high level of regular physical activity and exercise habits is strongly associated with a favorable clinical course in PD (Tsukita et al., 2022). The maximum duration of the research interventions included in this study was no more than 10 weeks (Pérez de la Cruz, 2017) and the minimum was 2 weeks (Vivas et al., 2011). Therefore, this may explain the lack of positive results in terms of motor function.

In terms of mobility, a sensitivity analysis was performed due to the high heterogeneity between the included studies. The results showed a positive long-term effect of hydrotherapy on mobility compared to the control group. The included literature was assessed for mobility using the TUG. TUG was used to assess balance and mobility in PD patients (Zampieri et al., 2010). It has been suggested that an improvement in balance also represents to some extent an improvement in mobility (Zhu et al., 2023). Therefore, this is one of the reasons why the sensitivity analysis yielded a positive result in terms of mobility.

## 5 Limitations

This systematic review has several limitations. First, too few studies were included in this meta-analysis. Second, only the long-term effects of hydrotherapy on motor symptoms in PD patients were explored; non-motor symptoms were not analyzed. Finally, because of the limitations of the literature that met the inclusion criteria, there was no subgroup analysis of the different hydrotherapy intervention regimens to determine the optimal hydrotherapy regimen for PD treatment.

## 6 Conclusion

The results of the meta-analysis showed that hydrotherapy had a positive maintenance effect on balance function in PD patients, but the long-term effects on motor function, mobility, and life therapy were not significant. Thus, hydrotherapy may be a highly effective and long-lasting form of physical therapy for PD patients who need to improve and maintain balance function.

## Data availability statement

The datasets presented in this study can be found in online repositories. The names of the repository/repositories and accession number(s) can be found in the article/supplementary material.

## Author contributions

ZL: Conceptualization, Data curation, Formal analysis, Methodology, Software, Writing – original draft, Writing – review & editing. MH: Funding acquisition, Resources, Visualization, Writing – original draft. YaL: Methodology, Project administration, Supervision, Validation, Writing – original draft. XX: Data curation, Formal analysis, Methodology, Project administration, Supervision, Validation, Writing – original draft. PZ: Writing – original draft, Writing – review & editing, Supervision, Validation. YanL: Supervision, Validation, Visualization, Writing – original draft, Writing – review & editing. CT: Conceptualization, Formal analysis, Project administration, Validation, Writing – original draft.
